# Combination of the saline-immersion technique and a new thin therapeutic endoscope for endoscopic submucosal dissection of a duodenal subepithelial tumor

**DOI:** 10.1055/a-2497-2386

**Published:** 2025-01-14

**Authors:** Kosei Hashimoto, Hisashi Fukuda, Toshihiro Fujinuma, Yoshie Nomoto, Edward J. Despott, Hironori Yamamoto

**Affiliations:** 1 12838 Department of Medicine, Division of Gastroenterology, Jichi Medical University, Shimotsuke, Japan; 2 171090 Royal Free Unit for Endoscopy, The Royal Free Hospital, University College London Institute for Liver and Digestive Health, London, United Kingdom of Great Britain and Northern Ireland

Few cases of endoscopic submucosal dissection (ESD) for duodenal subepithelial tumors (SELs) have been reported, particularly in the challenging area of the duodenal bulb just behind the pyloric ring. This region presents a narrow working space, and the thin submucosal layer complicates dissection between the SEL and the muscular layer. The recent introduction of a new thin therapeutic endoscope (EG-840TP; Fujifilm, Tokyo, Japan) holds promise for ESD in such confined spaces 1 2 3 4 . Despite its small diameter of 7.9 mm, this endoscope is equipped with a 3.2-mm accessory channel, a waterjet function, and an extended down angle of up to 160° ( Fig. 1 , Fig. 2 ), making it well suited for therapeutic procedures 1 .

**Fig. 1:**
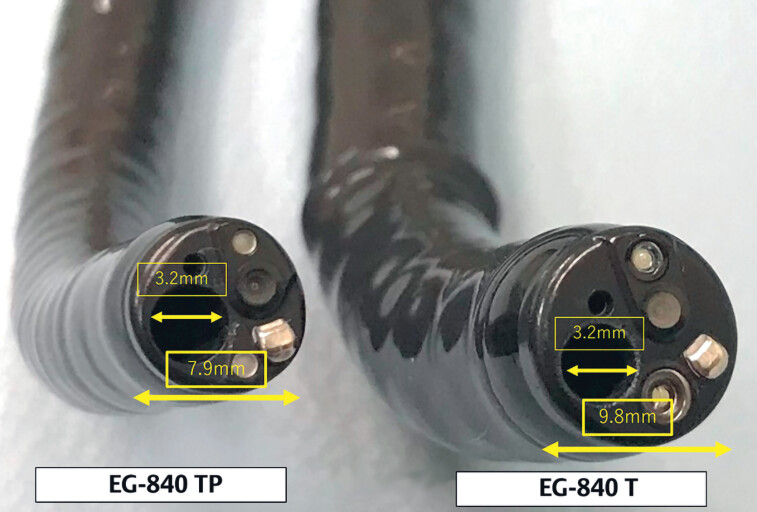
Photographic comparison of the newly developed thin therapeutic endoscope (EG-840TP; Fujifilm Co.) and a conventional therapeutic endoscope showing that both have an accessory channel with a diameter of 3.2 mm, but the new thin endoscope has an outer diameter of only 7.9 mm.

**Fig. 2:**
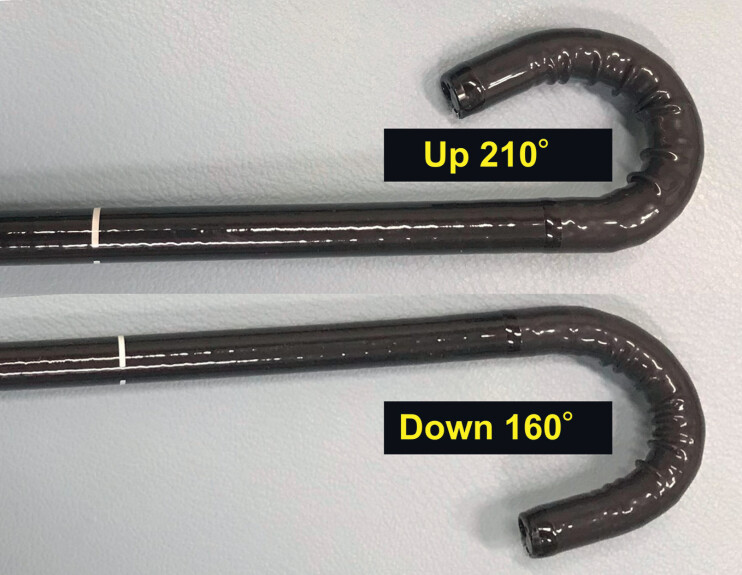
Photograph of the new thin therapeutic endoscope showing it has an extended down angle of 160°, while maintaining the up angle function at up to 210°.

We successfully performed total excision of a duodenal SEL with complete closure of the mucosal defect using this new thin endoscope and the saline-immersion technique for the ESD procedure ( Video 1 ). The patient was a 49-year-old man with an enlarging SEL in the duodenal bulb. Endoscopic ultrasound (EUS) revealed a 20-mm isoechoic mass originating from the second layer, with hypoechoic glandular structures inside, suggesting the possibility of an ectopic pancreas. Given the preservation of the third layer, ESD was deemed feasible. Although EUS-guided fine-needle aspiration (EUS-FNA) was considered, ESD was recommended owing to the diagnostic uncertainty and the potential need for long-term follow-up.

The combination of the saline-immersion technique and a new thin therapeutic endoscope is used to facilitate endoscopic submucosal dissection of a duodenal subepithelial tumor.Video 1

We employed a strategy to circumferentially incise the midportion of the elevation and dissect out the SEL along with the mucosa at the apex ( Fig. 3 ). Using the thin therapeutic endoscope fitted with a prototype transparent hood with a small-caliber tip, we easily approached the tumor, created a pocket from the oral side, and completed the resection within 40 minutes, with no adverse events occurring. The mucosal defect was easily closed with ordinary clips because enough room was left in the residual mucosa for closure. Pathological results confirmed the lesion to be Brunner’s gland hyperplasia measuring 25 × 20 mm.

**Fig. 3:**
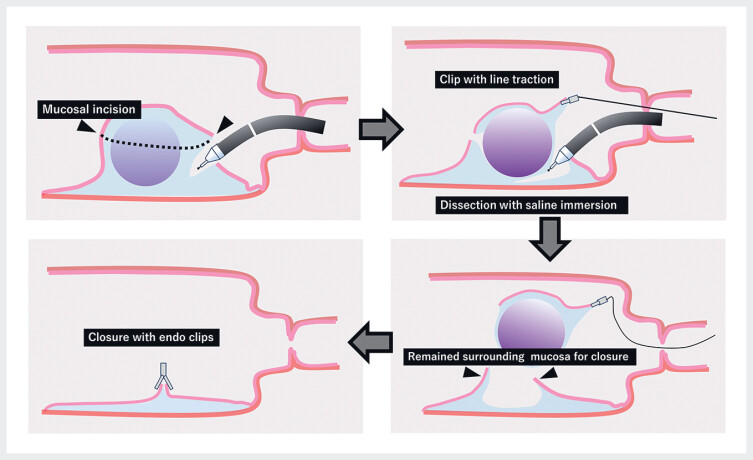
Schematic of the endoscopic submucosal dissection strategy for duodenal subepithelial tumors (SELs) using the combination of a new thin therapeutic endoscope with saline immersion and clip-with-line traction to facilitate safe and rapid dissection, which involved circumferential incision of the midportion of the elevation and dissection of the SEL together with the mucosa at the apex to facilitate subsequent closure of the mucosal defect.

Endoscopy_UCTN_Code_TTT_1AO_2AG_3AD

## Notes

Endoscopy E-Videos https://eref.thieme.de/e-videos
*E-Videos* is an open access online section of the journal *Endoscopy* , reporting on interesting cases and new techniques in gastroenterological endoscopy. All papers include a high-quality video and are published with a Creative Commons CC-BY license. Endoscopy E-Videos qualify for HINARI discounts and waivers and eligibility is automatically checked during the submission process. We grant 100% waivers to articles whose corresponding authors are based in Group A countries and 50% waivers to those who are based in Group B countries as classified by Research4Life (see: https://www.research4life.org/access/eligibility/ ). This section has its own submission website at https://mc.manuscriptcentral.com/e-videos .
